# Survival in elderly patients with breast cancer with and without autoimmune disease

**DOI:** 10.1002/cam4.5989

**Published:** 2023-04-26

**Authors:** Demitrios Dedousis, Annie L. Zhang, Anastasia N. Vassiliou, Shufen Cao, Deepthi Yammani, Ravi K. Kyasaram, John P. Shanahan, Melissa C. Keinath, Melinda L. Hsu, Pingfu Fu, Afshin Dowlati, Alberto J. Montero

**Affiliations:** ^1^ Department of Internal Medicine Case Western Reserve University and University Hospitals Seidman Cancer Center Cleveland Ohio USA; ^2^ Case Western Reserve University Cleveland Ohio USA; ^3^ Department of Population and Quantitative Health Sciences Case Western Reserve University Cleveland Ohio USA; ^4^ Case Western Reserve University and University Hospitals Seidman Cancer Center Cleveland Ohio USA; ^5^ Department of Pathology Case Western Reserve University and University Hospitals Seidman Cancer Center Cleveland Ohio USA; ^6^ Division of Hematology and Oncology Case Western Reserve University and University Hospitals Seidman Cancer Center Cleveland Ohio USA

**Keywords:** autoimmune diseases, breast neoplasms, Medicare; statistics & numerical data, prevalence, SEER program; statistics & numerical data, survival analysis

## Abstract

**Background:**

Patients with certain autoimmune conditions are at a reduced risk of developing breast cancer compared to the general population. Despite this, little is known about outcomes in patients with breast cancer who have a concurrent autoimmune diagnosis.

**Methods:**

This study compared differences in outcomes between women with breast cancer who had or did not have an autoimmune diagnosis. The SEER‐Medicare databases (2007–2014) were used to identify patients with breast cancer and diagnosis codes were used to identify those with an autoimmune disorder.

**Results:**

The studied autoimmune diseases had a prevalence of 27% among the 137,324 patients with breast cancer. Autoimmune disease was associated with significantly longer overall survival (OS) and significantly lower cancer‐specific mortality (CSM) among stage IV breast cancer patients (*p* < 0.0001). After controlling for the effects of age, race, chronic kideny disease, chemotherapy, and radiation therapy autoimmune disease was still predictive of improved OS (HR: 1.45, 95% CI: 1.35–1.55, *p* < 0.0001) and CSM (HR: 1.40, 95% CI: 1.29–1.5, *p* < 0.0001). By contrast, in patients with stage I–III breast cancer, the presence of an autoimmune diagnosis was associated with a lower OS (*p* < 0.0001, *p* < 0.0001, and *p* = 0.026, respectively), compared to patients without autoimmune disease.

**Conclusions:**

We found a higher prevalence of rheumatoid arthritis, Crohn's disease, ulcerative colitis, and systemic lupus erythematosus in patients with breast cancer compared to age matched cohorts in the general population. The presence of an autoimmune diagnosis was associated with a lower OS in stages I–III breast cancer and improved OS and CSM in patients with stage IV disease. These results suggest that anti‐tumor immunity plays an important role in late stage breast cancer and could potentially be exploited to improve the effectiveness of immunotherapy.

## Graphical abstract

This study investigated the impact of autoimmune disease on survival in women with breast cancer using a national database of cancer patients. Our analysis showed that women with breast cancer have higher rates of certain autoimmune conditions than previously reported including rheumatoid arthritis and inflammatory bowel disease. Patients with early stage breast cancer and autoimmune disease were found to have slightly worse survival, although this did not seem to be due to their breast cancer but instead may have been due to older age and a higher rate of kidney disease in those with an autoimmune diagnosis. However, we also found that in women with advanced breast cancer, having an autoimmune diagnosis, correlated with longer survival. This study raises the possibility that the immune system plays an important role in late stage breast cancer. More research is needed to clarify why autoimmune disease was linked to improved outcomes.

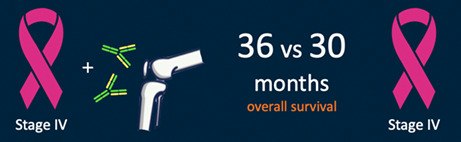

## 1 INTRODUCTION

The relationship between autoimmunity and cancer is complex. There are conflicting reports in the published literature reporting both improved and inferior outcomes in patients with cancer and a concomitant diagnosis of an autoimmune rheumatic disease, which suggests a dynamic and bidirectional relationship between the two.^1^, ^2^, ^3^, ^4^, ^5^, ^6^ Autoimmune diseases are a heterogeneous group of conditions that share a common mechanism involving autoantibodies or autoreactive T cells that attack “self” antigens leading to immune‐system‐mediated organ injury.^7^ Cancer mediated immune evasion is a hallmark of cancer.^8^ Therefore, overcoming cancer immune evasion, with the use of immune checkpoint inhibitors for example, has led to paradigm shifts in the treatment of many different cancers. Immunotherapy is also now being explored in traditionally “immunologically cold” malignancies like breast cancer highlighting the importance of studying the link between autoimmune disease and breast cancer.^9^

Several studies have shown a lower risk of developing breast cancer in patients with rheumatoid arthritis (RA) and systemic lupus erythematosus (SLE) compared to the general population.^1^, ^10^, ^11^, ^12^ Many potential explanations for this may be endocrine in nature as estrogen exposure is a well described risk factor for breast cancer^13^ and women with SLE undergo natural menopause at an earlier age.^14^ Treatment of autoimmune diseases may also influence cancer risk. There is some evidence that NSAIDs, often also used to treat rheumatic diseases, may reduce the risk of breast cancer.^15^ Another possible mechanism is the anti‐tumor effect of the autoimmune disease itself. Research has found that a naturally produced HER2 auto‐antibody that is protective against breast cancer^16^ and the presence of CD8+ T cells within breast tumors was associated with a 27% and 28% reduction in cancer mortality in ER+/HER2+ and ER‐ cancers, respectively.^17^ While the relationship between breast cancer incidence and autoimmune disease has been previously evaluated in a number of studies, only a few other studies have explored the impact of autoimmune disease on survival outcomes in breast cancer patients.^1^, ^2^, ^3^, ^4^, ^5^ Most of these studies found poorer survival in patients with autoimmune disease and breast cancer,^12^, ^13^, ^14^, ^15^ including specifically patients with early stage breast cancer.^6^ One notable exception was the study by Hemminki et al,^4^ which did not find any differences in overall survival (OS) in patients with breast cancer and a wide variety of autoimmune diseases, including RA. The objective of our study was to investigate the impact of a diagnosis of concomitant autoimmune disease on survival outcomes in patients with breast cancer.

## 2 MATERIALS AND METHODS

Materials and methods were adapted from a previous study also published by this research group.^18^

### 2.1 Data source

SEER‐Medicare^19^ is a linked national cancer registry that includes adults ≥65 years old or adults with end stage renal disease or disability residing within SEER survey areas, representing roughly 25% of the US population.^20^ This linked database includes information on patient demographics, cancer‐specific data, and Medicare claims data. In this study, SEER‐Medicare patients diagnosed with breast cancer from 2007–2014 were examined. ICD‐9 diagnosis codes were used to identify patients with common autoimmune diseases (Table S1). Chronic condition flags were also used to identify those with RA. To be included, patients needed to be labeled with one or more relevant ICD‐9 codes or chronic condition flags either during a 2‐year lookback period prior to the breast cancer diagnosis, or after the date of cancer diagnosis. The vast majority (87%) of patients included in the autoimmune cohort had their first autoimmune diagnosis code applied either up to 2 years before their cancer diagnosis (16%) or concurrently with the breast cancer diagnosis (71%). A minority (13%) of patients had their first autoimmune diagnosis code applied after their cancer diagnosis; the median duration between breast cancer diagnosis and autoimmune diagnosis was approximately 2 years. The following autoimmune diseases were included in our investigation: Crohn's disease (CD), dermatomyositis, polymyositis, psoriasis, RA, sarcoidosis, systemic scleroderma, Sjogren's syndrome, SLE, and ulcerative colitis (UC). Common autoimmune diseases were drawn from a standard medical text.^21^ The autoimmune diseases included in this analysis were then limited to those with ICD codes that could identify patients specifically affected with these conditions (Table S1), and which also had at least 100 patients included in the overall cohort.^22^ ICD‐10 diagnosis codes were used to identify those whose deaths were caused by their breast cancer (Table S2).

Baseline characteristics included age, sex, race, level of urban development, poverty rates within the census tract, chronic kidney disease (CKD), hormone receptor status, breast cancer stage, and oncologic treatment history including chemotherapy and any surgery or radiation treatment within 4 months of cancer diagnosis. A diagnosis of CKD by SEER‐Medicare required at least one or two (depending on the type of claim) insurance claims with a CKD diagnosis code.^23^ Breast cancer stage was defined according to the American Joint Committee on Cancer (AJCC) Cancer Staging Manual, Sixth^16^ and Seventh Editions.^17^ The specific chemotherapy drugs used in this cohort were identified using Healthcare Common Procedure Coding System (HCPCS) codes (Table S3). A secure HIPPA‐compliant server stored this study's research data. The server is protected, by the Seidman Cancer Center at University Hospitals Cleveland Medical Center's firewall, and requires continuous logging and access controls.

### 2.2 Exclusion criteria

**FIGURE 1:**
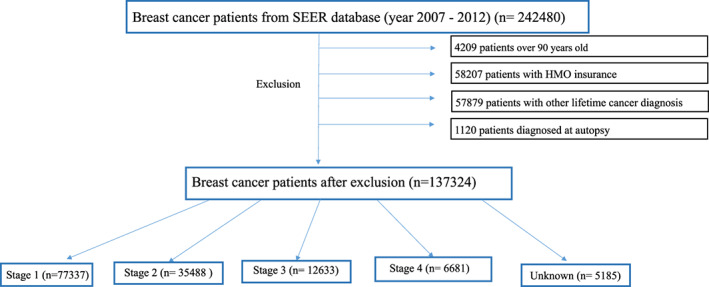
Consort flow diagram showing the selection of eligible breast cancer patients including specific exclusions from the SEER Medicare Database (2007–2014) and breakdown of number of patients by disease stage. HMO, health maintenance organization.

Certain patients were excluded from the analysis: (1) individuals whose age was ≥90 years, as recorded on the date of breast cancer diagnosis; (2) individuals with health maintenance organization (HMO) insurance; (3) patients with an additional cancer diagnosis of any kind during their lifetime; and (4) those first identified as having breast cancer during autopsy (Figure 1).

### 2.3 Primary outcomes

This study has two co‐primary endpoints: OS and cancer‐specific mortality (CSM). Death from a non‐cancer related cause was a competing risk. OS was defined as the time that elapsed between the date of breast cancer diagnosis and date of death. OS was censored for survivors at the date of last recorded follow up. CSM was defined as the time that elapsed between the date of cancer diagnosis and the date of death from a non‐cancer cause. CSM was censored for those still alive at the date of last recorded follow up.

### 2.4 Statistical analysis

Descriptive statistics were used to assess baseline patient and cancer characteristics, additionally the chi square test with continuity correction was used as the test of significance for differences in categorical variables between the groups of patients who have and do not have an autoimmune diagnosis. The Kaplan‐Meier method was used to estimate the OS with log rank test used for the difference between groups.^24^ CSM was estimated taking non‐cancer death as competing risk into account.^25^, ^26^ Multivariable Cox regression was employed to further estimate the effect of autoimmune disease on OS by controlling for age, race, CKD, chemotherapy, and radiation therapy.^27^ Gray's method^26^ was used to further clarify the effect of autoimmune disease on CSM adjusting for the effects of age, race, CKD, and oncologic treatment. All tests of significance to evaluate OS and CSM were two‐sided and *p*‐values ≤0.05 were considered statistically significant. Analyses were conducted using R statistical software (version 4.1.3; The R Foundation) and SAS statistical software (version 9.4; SAS Institute Incorporated).

## 3 Selected results

### 3.1 Population characteristics and autoimmune disease prevalence

Patients with breast cancer (*n* = 242,480) were identified from the SEER‐Medicare database. A total of 105,156 patients did not meet eligibility criteria and were excluded, the remaining 137,324 patients were included in the analysis (Figure 1). Most, 56% (*n* = 77,337), had stage I disease. Approximately 26% (*n* = 35,488), 9% (*n* = 12,633), and 5% (*n* = 6681) had stage II–IV disease, respectively, while in approximately 4% of patients (*n* = 5185) stage was unknown. The overall prevalence of the included autoimmune diagnoses in the 137,324 eligible patients was approximately 27%. Table 1 lists the prevalence of each included autoimmune disease; with RA (23.35%), psoriasis (2.41%), and SLE (1.12%) being the most prevalent.

**TABLE 1:** Frequency of autoimmune disease in breast cancer cohort (*n* = 36,647) compared to frequency in general population.

Autoimmune disease	Frequency (%)	General population estimate %
Crohn's disease	976 (0.71)	0.21^29^
Dermatomyositis	152 (0.11)	n/a^a^
Polymyositis	205 (0.15)	n/a^a^
Psoriasis	3305 (2.41)	1.13^33^, ^b^
RA	32,068 (23.35)	2.35–2.71^28^
Sarcoidosis	566 (0.41)	0.06^31^, ^c^
Scleroderma	370 (0.27)	0.02–0.05^39^, ^40^, ^c^
Sjogren's syndrome	1231 (0.9)	n/a^a^
SLE	1540 (1.12)	0.37^30^
UC	1504(1.1)	0.32^29^

*Note*: Frequency of autoimmune diseases in breast cancer by individual disease (*n* = 137,324) and estimates of prevalance from age (and for RA sex) matched cohorts without cancer for comparison. Overall prevalence of autoimmune disease was 27% (36,647/137,324).

Abbreviations: n/a, not applicable; RA, rheumatoid arthritis; SLE, systemic lupus erythematosus; UC, ulcerative colitis.

^a^ Insufficient high quality prevalence data available.

^b^ Estimate based on data from 1 year.

^c^ Includes age > 18.

Patient characteristics which included: sex, race, urban versus rural locality, and poverty rates, were numerically similar when comparing the cohorts of patients with and without an autoimmune diagnosis (Table 2). As expected, nearly all the patients in this cohort (>99%) were women. Most patients in the overall cohort self‐reported as White (81%). In the entire cohort, approximately 11% self‐reported as Black, however, among patients with stage IV disease the proportion of Black women was even higher at 15%. Most patients lived in urban or mostly urban areas (86%), and only a minority lived in rural or mostly rural areas (14%). Most patients (81%) lived in municipalities with 0%–20% of residents living in poverty, 19% of patients live in municipalities with >20% of residents living in poverty. Patients in the autoimmune diagnosis group were older than those without a history of autoimmune disease (68 vs. 62 years, *p* < 0.001). This was also seen but was less pronounced in the stage IV breast cancer cohort (69 vs. 66, *p* < 0.001). Disease stage at the time of diagnosis did differ significantly between both groups (*p* < 0.001), but absolute differences on a percentage basis were small. A similar trend was also noted with both hormone receptor and HER2 status (all *p* values, *p* < 0.001). Patients with an autoimmune diagnosis were more likely to have CKD (27% vs. 14%, *p* < 0.001). The difference in the prevalence of CKD was even larger in those with stage IV breast cancer (43% vs. 25%, *p* < 0.001). The percentages of those treated with radiation were similar in both groups. Significantly fewer women with an autoimmune diagnosis were treated with surgery (*p* < 0.001); however, this difference was small. With respect to chemotherapy, patients with autoimmune disease in the overall cohort were significantly more likely to have received chemotherapy (18% vs. 10%, *p* < 0.001). This difference was even more pronounced in patients with stage IV breast cancer (41% vs. 27%, *p* < 0.001).

**TABLE 2:** Patient characteristics (*n* = 137,324).

Variables	Median (range) or frequency (%)								
	Autoimmmune disease breast cancer cohort			Autoimmune disease stage I–III breast cancer			Autoimmmune disease stage IV breast cancer		
	No (*n* = 100,677)	Yes (*n* = 36,647)	*p* value	No (*n* = 91,526)	Yes (*n* = 33,932)	*p* value	No (*n* = 5330)	Yes (*n* = 1351)	*p* value
Age (range)	62 (58, 72)	68 (63, 75)	<0.001	62 (21, 90)	68 (23, 90)	<0.0001	66 (58, 76)	69 (64, 77)	<0.001
Sex (%)			<0.001			0.0004			1
Female	99,877 (99.2)	36,425 (99.4)		90,830 (99.2)	33,738 (99.4)		5268 (98.8)	1335 (98.8)	
Male	800 (0.8)	222 (0.6)		696 (0.8)	194 (0.6)		62 (1.2)	16 (1.2)	
Race (%)			<0.001			<0.0001			0.005
Black	10,842 (10.8)	3875 (10.6)		9573 (10.5)	3492 (10.4)		805 (15.1)	211 (15.6)	
White	81,486 (80.9)	30,757 (83.9)		74,292 (81.6)	28,577 (84.6)		4195 (78.7)	1090 (80.7)	
Others	8349 (8.3)	2015 (5.4)		7209 (7.9)	1700 (5)		330 (6.2)	50 (3.7)	
Rural/Urban (%)			<0.001			<0.0001			0.612
All urban	65,815 (65.4)	23,309 (63.6)		59,975 (65.5)	21,547 (63.5)		>3400 (>63.8)^a^	>850 (>62.9)^a^	
Mostly urban	21,074 (20.9)	8102 (22.1)		19,143 (20.9)	7551 (22.2)		1116 (20.9)	271 (20.1)	
Mostly rural	7158 (7.1)	2659 (7.3)		6482 (7.1)	2485 (7.3)		375 (7.0)	92 (6.8)	
All rural	6483 (6.4)	2506 (6.8)		5815 (6.4)	2299 (6.8)		389 (7.3)	89 (6.6)	
Unknown	147 (0.1)	71 (0.2)		111 (0.1)	50 (0.2)		<50 (<0.9)^a^	<50 (<3.7)^a^	
Poverty (%)			<0.001			<0.0001			0.147
0%–5%	24,547 (24.4)	9003 (24.6)		22,627 (24.7)	8451 (24.9)		1081 (20.3)	280 (20.7)	
5%–10%	27,652 (27.5)	9817 (26.8)		25,361 (27.7)	9117 (26.9)		1319 (24.7)	366 (27.1)	
10%–20%	28,965 (28.8)	10,300 (28.1)		26,232 (28.7)	9539 (28.1)		>1600 (>30.0)^a^	>350 (>26.0)^a^	
20%–100%	19,348 (19.2)	7451 (20.3)		17,171 (18.8)	6767 (19.9)		1315 (24.7)	334 (24.7)	
Unknown	165 (0.2)	76 (0.2)		135 (0.1)	58 (0.2)		<15 (<0.3)^a^	<21 (<1.6)^a^	
ER (%)			<0.001			<0.0001			0.407
Positive	76,573 (76.1)	28,370 (77.4)		71,011 (77.6)	26,677 (78.6)		>3550 (>66.6)^a^	>900 (>66.6)^a^	
Negative	16,319 (16.2)	53,70 (14.7)		14,816 (16.2)	4932 (14.5)		1027 (19.3)	259 (19.2)	
Bordeline	118 (0.1)	36 (0.1)		103 (0.1)	31 (0.1)		<37 (<0.7)^a^	<28 (<2.1)^a^	
Unknown	7667 (7.6)	2871 (7.8)		5596 (6.1)	2292 (6.8)		716 (13.4)	164 (12.1)	
PR (%)			<0.001			<0.0001			0.791
Positive	64,396 (64.0)	24,138 (65.9)		59,965 (65.5)	22,769 (67.1)		2778 (52.1)	>700 (>51.8)^a^	
Negative	27,084 (26.9)	9204 (25.1)		24,547 (26.8)	8475 (25)		1766 (33.1)	435 (32.2)	
Bordeline	362 (0.4)	126 (0.3)		314 (0.3)	114 (0.3)		25 (0.5)	<21 (<1.6)^a^	
Unknown	8835 (8.8)	3179 (8.7)		6700 (7.3)	2574 (7.6)		761 (14.3)	185 (13.7)	
HER2/HR (%)			<0.001			<0.0001			0.001
HER2+/HR+	4030 (4.0)	1115 (3.0)		3478 (3.8)	997 (2.9)		427 (8.0)	74 (5.5)	
HER2+/HR‐	1827 (1.8)	482 (1.3)		1572 (1.7)	422 (1.2)		202 (3.8)	40 (3.0)	
HER2‐/HR+	29,002 (28.8)	9184 (25.1)		26,923 (29.4)	8604 (25.4)		1404 (26.3)	343 (25.4)	
Triple Negative	4221 (4.2)	1258 (3.4)		3892 (4.3)	1146 (3.4)		229 (4.3)	77 (5.7)	
Unknown	61,597 (61.2)	24,608 (67.1)		55,661 (60.8)	22,753 (67.1)		3068 (57.6)	817 (60.5)	
CKD (%)			<0.001			<0.0001			<0.001
No	86,239 (85.7)	26,753 (73.0)		79,334 (86.7)	25,044 (73.8)		3984 (74.7)	767 (56.8)	
Yes	14,438 (14.3)	9894 (27.0)		12,192 (13.3)	8888 (26.2)		1346 (25.3)	584 (43.2)	
CTX (%)			<0.001			<0.0001			<0.001
No	90,737 (90.1)	29,878 (81.5)		83,499 (91.2)	28,014 (82.6)		3883 (72.9)	801 (59.3)	
Yes	9940 (9.9)	6769 (18.5)		8027 (8.8)	5918 (17.4)		1447 (27.1)	550 (40.7)	
Surgery (%)			<0.001			<0.001			0.3
No	8486 (8.4)	2486 (6.8)		2911 (3.2)	957 (2.8)		3546 (66.5)	878 (65.0)	
Yes	92,191 (91.6)	34,161 (93.2)		88,615 (96.8)	32,975 (97.2)		1784 (33.5)	473 (35.0)	
Radiation (%)			0.114			0.0002			0.275
No	49,970 (49.6)	18,416 (50.3)		43,141 (47.1)	16,400 (48.3)		3667 (68.8)	908 (67.2)	
Yes	50,707 (50.4)	18,231 (49.7)		48,385 (52.9)	17,532 (51.7)		1663 (31.2)	443 (32.8)	
Stage (%)			<0.001						
1	55,870 (55.5)	21,467 (58.6)							
2	26,035 (25.9)	9453 (25.8)							
3	9621 (9.6)	3012 (8.2)							
4	5330 (5.3)	1351 (3.7)							
Unknown	3821 (3.8)	1364 (3.7)							

*Note*: Characteristics of patients with breast cancer (*n* = 137,324), stage I‐III breast cancer (*n* = 125,458) and stage IV breast cancer (*n* = 6681) with and without autoimmune diagnosis.

Abbreviations: CKD, chronic kidney disease; CTX, chemotherapy; ER, estrogen receptor; HER2+, human epidermal growth factor receptor 2 positive; HR+, hormone receptor positive; PR, progesterone receptor.

^a^ Data in cell coarsened as per SEER‐Medicare data use agreement to protect patient identity.

### 3.2 Survival analysis

**FIGURE 2:**
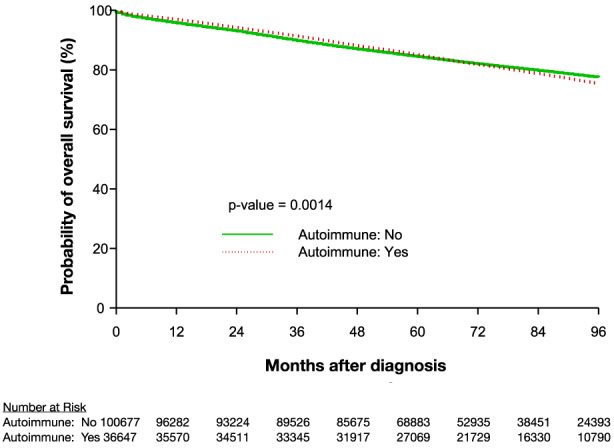
Kaplan–Meier estimation of OS for patients with breast cancer with and without an autoimmune diagnosis. OS, overall survival.

**FIGURE 3:**
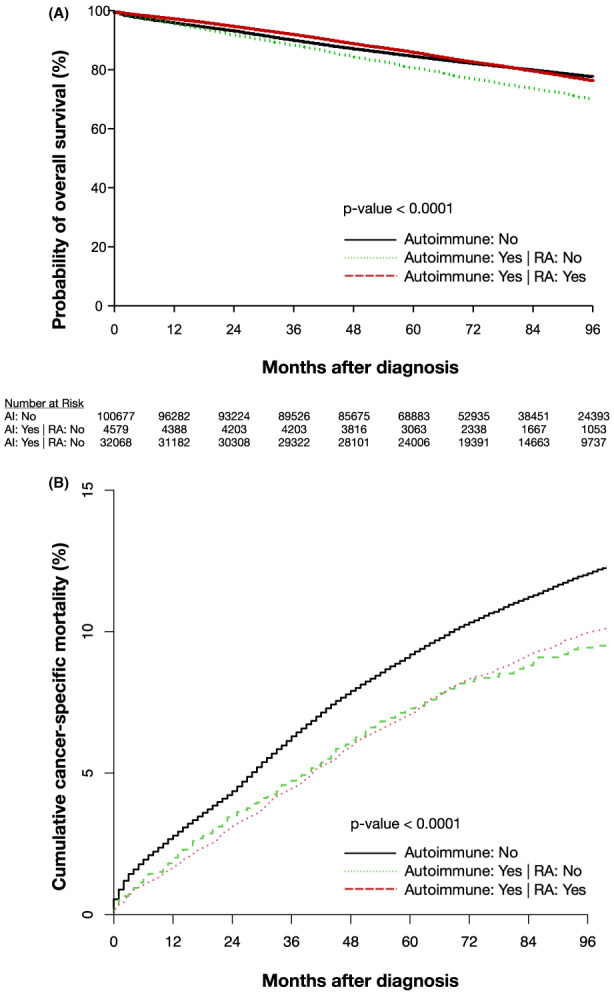
(A) Kaplan‐Meier estimation of OS for patients with breast cancer with RA, with autoimmune diagnosis other than RA or without an autoimmune diagnosis. (B) Cumulative incidence of CSM for patients with breast cancer with RA, with autoimmune diagnosis other than RA or without autoimmune diagnosis. CSM, cancer‐specific mortality; OS, overall survival; RA, rheumatoid arthritis.

In the overall cohort, patients with breast cancer who also had a known autoimmune diagnosis had a longer OS (*p* = 0.0014), which was statistically significant when compared to patients without an autoimmune diagnosis, however, the difference was very modest and the survival curves were almost completely overlapping (Figure 2). The median overall survival (mOS) was not reached for either subgroup. In patients with breast cancer who also had RA, we observed significant OS differences (*p* < 0.0001) between them and those, with a diagnosis of a different non‐RA autoimmune diagnosis, and patients without a diagnosis of an autoimmune disease (Figure 3A). The difference in OS between those with RA and those without an autoimmune diagnosis was small with mostly overlapping OS curves. Patients with breast cancer who also had a diagnosis of RA, had significantly longer OS than patients with a diagnosis of a different autoimmune disease. There was also a significant difference in CSM between the 3 groups (*p* < 0.0001), with CSM being lower in the patients with a diagnosis of an autoimmune disease, whether RA or a different autoimmune diagnosis, than in patients without an autoimmune diagnosis. There was not a statistically significant difference in CSM between the two autoimmune subsets, RA versus other autoimmune diagnosis (*p* = 0.536; Figure 3B).

**FIGURE 4:**
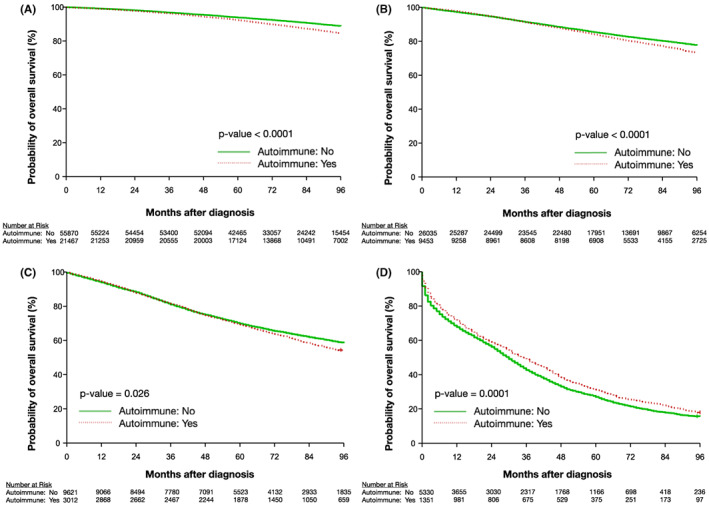
Kaplan‐Meier estimation of OS for patients with breast cancer with and without an autoimmune diagnosis by breast cancer stage: (A) stage I (B) stage II (C) stage III (D) stage IV. OS, overall survival.

**FIGURE 5:**
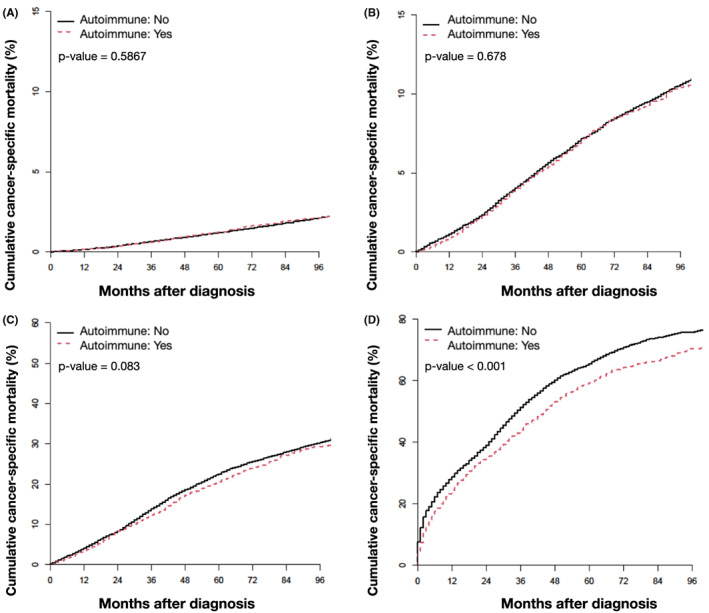
Cumulative incidence of CSM for patients with breast cancer with and without an autoimmune diagnosis by breast cancer stage: (A) stage I (B) stage II (C) stage III (D) stage IV. CSM, cancer specific mortality.

Patients with stage I–III breast cancer and an autoimmune diagnosis, were found to have a significantly worse OS (*p* < 0.0001, *p* < 0.0001, and *p* = 0.026, respectively) than patients without an autoimmune diagnosis, with the greatest separation of OS curves occurring after 60 months (Figure 4A–C). The mOS was not reached in patients with stage I‐II breast cancer, in both the autoimmune and non‐autoimmune diagnosis groups. Among patients with stage III breast cancer without an associated autoimmune diagnosis, the mOS was not reached; among patients with an associated autoimmune diagnosis and stage III breast cancer the mOS was 113 months. After adjusting for age, race, CKD, chemotherapy, and radiation therapy, the presence of an autoimmune diagnosis was predictive of a significantly improved OS in patients with stage I–III breast cancer (hazard ratio (HR): 1.05, 95% confidence interval (CI): 1.01–1.10, *p* = 0.25, HR: 1.24, 95% CI: 1.18–1.31, *p* < 0.0001, HR: 1.34, 95% CI: 1.25–1.43, *p* < 0.0001, respectively; Table S4A–S6A). Among patients with stage I–III breast cancer, CSM did not significantly differ by the presence or absence of an autoimmune diagnosis (*p* = 0.587, *p* = 0.678, *p* = 0.083, respectively) (Figure 5A–C). However, after controlling for age, race, CKD, and oncologic treatment, an autoimmune diagnosis was associated with a lower CSM (HR: 1.35, 95% (CI): 1.20–1.51, *p* < 0.0001, HR: 1.36, 95% CI: 1.26–1.47, *p* < 0.0001, HR: 1.41, 95% CI: 1.29–1.53, *p* < 0.0001, respectively) (Table S4B–S6B).

By contrast, among patients with stage IV breast cancer, we observed that an autoimmune diagnosis was correlated with a significantly higher OS (Figure 4D), and a significantly lower CSM (*p* < 0.0001) (Figure 5D). The estimated mOS in the patients with stage IV breast cancer and an autoimmune diagnosis was 36 months versus 30 months for patients with stage IV breast cancer who did not have a documented autoimmune diagnosis. Even after adjusting for the effects of age, race, CKD, and oncologic treatment, an autoimmune diagnosis was still predictive of improved OS (HR: 1.45, 95% CI: 1.35–1.55, *p* < 0.0001) and CSM (HR: 1.40, 95% CI: 1.29–1.50, *p* < 0.0001; Tables S7A,B).

In the entire breast cancer cohort, certain baseline patient characteristics were correlated with OS. After adjusting for the impact of the following factors: autoimmune diagnosis, race, CKD, chemotherapy, and radiation therapy, each year of additional age was associated with a lower OS across all stages (I, HR: 1.09, 95% (CI): 1.09–1.10, *p* < 0.0001; II, HR: 1.06, 95% CI: 1.06–1.06, *p* < 0.0001; III, HR: 1.03, 95% CI: 1.03–1.04, *p* < 0.0001; IV, HR: 1.036, 95% CI: 1.03–1.04, *p* < 0.0001). Patients with breast cancer who self‐identify as Black had significantly worse OS than patients self‐identifying as White across all stages, even after adjusting for the impact of an autoimmune diagnosis, age, CKD and oncologic treatment, (I, HR: 1.33, 95% (CI): 1.23–1.43, *p* < 0.0001; II, HR: 1.34, 95% CI: 1.25–1.43, *p* < 0.0001; III, HR: 1.42, 95% CI: 1.32–1.54, *p* < 0.0001; IV, HR: 1.32, 95% CI: 1.23–1.43, *p* < 0.0001). We also observed that patients with breast cancer who self‐identified as other than Black or White had significantly improved OS compared to patients who self‐identified as White for all stages (I, HR: 0.58, 95% (CI): 0.51–0.65, *p* < 0.0001; II, HR: 0.73, 95% CI: 0.66–0.82, *p* < 0.0001; III, HR: 0.86, 95% CI: 0.75–0.97, *p* = 0.018; IV, HR: 0.72, 95% CI: 0.63–0.82, *p* < 0.0001). CKD was predictive of lower OS even when age, race, autoimmune disease and oncologic treatment are adjusted for across all stages (I, HR: 0.35, 95% (CI): 0.33–0.36, *p* < 0.0001; II, HR: 0.39, 95% CI: 0.38–0.40, *p* < 0.0001; III, HR: 0.47, 95% CI: 0.44–0.50, *p* < 0.0001; IV, HR: 0.76, 95% CI: 0.71–0.8, *p* < 0.0001) (Tables S4A–S7A).

## 4 DISCUSSION

This study used a national cohort to investigate the impact of common autoimmune disorders on breast cancer survival outcomes. Our survival analysis showed a statistically significant improvement in OS among patients with breast cancer and a diagnosis of an autoimmune disease compared to those without autoimmune disease. This improvement in OS was primarily driven by patients with an RA diagnosis who were found to have a significantly higher OS, compared both to patients with a different non‐RA autoimmune diagnosis and patients without an autoimmune diagnosis. Patients with a different non‐RA autoimmune diagnosis had lower OS than patients without an autoimmune diagnosis. Given that patients with a diagnosis of an autoimmune disease other than RA had a significantly lower CSM than patients without any autoimmune diagnosis, the lower OS in patients with a non‐RA autoimmune diagnosis is likely not cancer related, and is possibly due to differences in baseline characteristics such as older age and higher rates of CKD among patients with autoimmune disease.

In patients with non‐metastatic breast cancer (stages I–III) we observed that an autoimmune diagnosis was associated with worse OS. However, in these patients we observed no difference in CSM in those with or without an autoimmune diagnosis. Furthermore, when controlling for differences in age, race, CKD, chemotherapy, and radiation therapy, we found that an autoimmune diagnosis was actually associated with a significantly improved OS compared to patients with non‐metastatic breast cancer without an autoimmune diagnosis. Interestingly, in patients with stage IV breast cancer, we found the inverse relationship; a diagnosis of an autoimmune disorder was correlated with significantly improved OS and a mOS that was approximately 6 months longer than patients with stage IV breast cancer without an autoimmune diagnosis. This improved OS was at least in part due to a reduction in CSM associated with an autoimmune diagnosis and was not wholly explained by differences in baseline patient characteristics.

The improvement in OS identified in this study in patients with breast cancer and an autoimmune diagnosis both in the overall cohort, among patients with RA specifically and in stage IV breast cancer contrasts with most previously reported studies.^12^, ^13^, ^14^, ^15^ However this study's findings showing reduced OS in stage I‐II breast cancer agreed with existing literature.^4^, ^6^ A study using an older SEER‐Medicare cohort (1992–2000) found significantly reduced OS in patients with breast cancer and RA, including in stage I‐II disease. However, patients with stage III–IV disease and RA, who were grouped together, did not have significantly worse OS than those without RA. Another study using the SEER‐Medicare cohort and Texas Cancer Registry which specifically looked at SLE also found reduced survival in patients with autoimmune diseases and stage I‐II breast cancer.^6^ A study using the Texas Cancer Registry alone found a significant reduction in survival in patients with RA and breast cancer even when stage was adjusted for.^13^ One study using a Swedish national cohort showed worse OS and CSM in patients with both RA and breast cancer.^5^ Notably, that study used a cohort with limited data on disease stage and therefore could not breakdown cohorts of patients with cancer by stage possibly masking an improvement in stage IV disease. It also used hospitalization for RA as its criteria to identify patients with RA thus likely identifying patients with more severe or poorly controlled RA possibly exaggerating the negative effects RA had on survival. Finally, another study using a Swedish national cohort and looking at a wide variety of autoimmune diseases did not find a significant difference in survival between patients with breast cancer with and without autoimmune disease. That study also did not find a significant difference in survival when looking specifically at RA.^4^

The overall prevalence of autoimmune disease in this population was approximately 27%. Several autoimmune diagnoses were enriched in this breast cancer cohort (Table 1). Most notably, the prevalence of RA among breast cancer patients was more than 8 times greater than the prevalence among females over the age of 60 in the United States (23% vs. 3%, respectively).^28^ The prevalence of UC and CD were also greater in the breast cancer cohort compared to age matched cohorts in the general population (UC: 1.1% vs. 0.32%; CD: 0.71% vs. 0.21%).^29^ A higher prevalence for a diagnosis of SLE was also observed among breast cancer patients (1.12% vs. 0.4%). Using prevalence data for adults older than 18 as a comparator, there is enrichment of sarcoidosis (0.41% vs. 0.05%), and scleroderma (0.27% vs. 0.05%) in the studied cohort.^30^, ^31^, ^32^ Data on the prevalence of psoriasis is limited to a study analyzing Medicare claims from one single year. Breast cancer patients in this cohort had a prevalence of psoriasis of 2.41% compared to 1.13% as described in Takeshita et al.^33^ We excluded comparison with dermatomyositis, polymyositis, and Sjogren's syndrome due to insufficient high‐quality prevalence data.^34^, ^35^ While there may be a biologic explanation for higher rates of autoimmune disease found among breast cancer patients, it is important to note methodological differences in estimating prevalence. For example, Rasch et al. relied on clinical criteria to identify RA whereas our study used diagnosis codes.^28^

There were several notable findings in terms of baseline patient characteristics of this cohort related to race and CKD. Whites were overrepresented in this breast cancer cohort compared to the general population of the United States (81% vs. 72%) while Blacks were underrepresented in this cohort compared to the general population (11% vs. 13%).^36^ The kidney damage caused by diseases such as SLE could explain the elevated rates of CKD among those with autoimmune disease. Furthermore, patients on dialysis, even those younger than 65 years of age, are eligible for Medicaid enrollment which may partially explain the relatively high frequency of CKD in both those who had and did not have an autoimmune diagnosis, 27% and 14%, respectively.^37^ Among patients with metastatic breast cancer the presence of CKD, a significant additional comorbidity, was predictive of lower OS even when autoimmune disease, age, race, chemotherapy, and radiation therapy are controlled for.

The large size of this cohort decreases the possibility that the observed differences in OS are due to random chance. The 7‐year period data was collected over allowed for long term follow up. Furthermore, the years included in this study predate the use of immune checkpoint inhibitors in breast cancer, removing a possible confounder, and isolating the effect of autoimmune disease on outcomes from that of treatment with immunotherapy. There were also certain limitations to this analysis. SEER‐Medicare data largely includes patients >65 years old. Therefore, the findings of this study may not be generalizable to a younger population with breast cancer. Using diagnosis codes to identify patients with autoimmune disease could potentially allow patients that do not meet clinical and laboratory criteria for an autoimmune diagnosis to be included in the autoimmune group. Similarly, those with a true history of autoimmune disease that was not captured by the SEER‐Medicare database may not have been included. A small percentage of patients in the autoimmune cohort (13%) had their earliest autoimmune diagnosis code applied after their cancer diagnosis. This raises the possibility of survivor bias, longer lived patients are more likely to have the opportunity to develop new conditions. However, in these patients who had an autoimmune disease that developed or came to medical attention relatively soon after their cancer diagnosis, the autoimmune process could reasonably have influenced the disease course. It is difficult to discern if an autoimmune disease diagnosed after a cancer diagnosis was simply not documented until the patient was receiving closer monitoring due to their cancer treatment or if the patient truly did not develop an autoimmune disease until they reached advanced age.

Furthermore, the Charlson comorbidity and National Cancer Institute indices were excluded from the multivariate regressions due to collinearity between comorbidity indices and autoimmune disease (Tables S4A–S7B). Both indices include “rheumatologic conditions”, which makes interpreting the significance of a difference in either of the indices between the autoimmune and non‐autoimmune populations difficult. The SEER‐Medicare database does not include data on risk factors,^38^ meaning that tobacco use in this study population cannot be determined with any reliability. Therefore, excluding a difference in the prevalence of tobacco usage as an underlying driver of survival differences between the patients with and without an autoimmune diagnosis is difficult.

## 5 CONCLUSIONS

This study observed a higher prevalence of specific common autoimmune diseases such as RA, CD, UC, and SLE in patients with breast cancer compared to cohorts of similar age ranges in the general population. A diagnosis of an autoimmune disease in patients with stage I–III breast cancer was associated with a significantly worse OS, which disappeared when controlling for differences in age, race, CKD, chemotherapy, and radiation therapy. Moreover, in patients with non‐metastatic breast cancer the presence of an autoimmune diagnosis was not associated with any significant differences in CSM. By contrast, in patients with advanced stage breast cancer, a diagnosis of an autoimmune disease was associated with significantly improved OS and CSM even when controlling for differences in age, race, CKD, and oncologic treatments. This study raises the possibility that anti‐tumor immunity plays an important role in late stage breast cancer. Additional investigation is required to further elucidate the interplay between autoimmune diseases and breast cancer; as well as to explore the relationship between autoimmune disorders and outcomes in other cancer types. Pathways found to be contributing to the development of an elevated antitumor immune response in patients with autoimmune disease could be exploited to improve the effectiveness of immunotherapy and for future drug development.

## AUTHOR CONTRIBUTIONS

**Demitrios Dedousis:** Conceptualization (lead); data curation (equal); formal analysis (lead); investigation (lead); methodology (lead); project administration (lead); supervision (equal); validation (lead); visualization (equal); writing – original draft (lead); writing – review and editing (equal). **Annie L Zhang:** Project administration (equal); validation (equal); visualization (equal); writing – original draft (equal); writing – review and editing (equal). **Anastasia N Vassiliou:** Data curation (equal); formal analysis (equal); investigation (equal); software (equal); visualization (equal); writing – original draft (equal). **Shufen Cao:** Data curation (equal); formal analysis (equal); methodology (equal); software (equal); validation (equal); visualization (equal); writing – original draft (equal). **Deepthi Yammani:** Data curation (equal); formal analysis (equal); software (equal); validation (equal). **Ravi K. Kyasaram:** Data curation (supporting); software (supporting). **John Shanahan:** Resources (equal); supervision (supporting); writing – original draft (supporting). **Melissa C Keinath:** Visualization (equal). **Melinda L Hsu:** Supervision (equal); writing – review and editing (equal). **Pingfu Fu:** Data curation (equal); formal analysis (equal); methodology (equal); resources (equal); software (equal); supervision (equal); validation (equal); visualization (equal). **Afshin Dowlati:** Conceptualization (equal); formal analysis (equal); funding acquisition (lead); methodology (equal); resources (equal); supervision (equal); validation (equal); writing – review and editing (equal). **Alberto J Montero:** Conceptualization (equal); supervision (lead); validation (equal); writing – review and editing (equal).

## FUNDING INFORMATION

This work was supported by the Division of Hematology and Oncology at University Hospitals.

## CONFLICT OF INTEREST STATEMENT

Dr. Dowlati reports personal fees from Seattle Genetics, AstraZeneca, Jazz Pharmaceuticals, Bristol Myers Squibb, Ipsen, Eli Lilly, Merck, Abbvie, and G1 Therapeutics outside the submitted work. The remaining authors declare no conflict of interest.

## ETHICS APPROVAL STATEMENT

This study was reviewed by the University Hospitals Institutional Review Board and determined to be an exempt study. All standard ethical guidelines were followed.

## Supporting information


Data S1:

## Data Availability

The datasets used for the current study are available from SEER‐Medicare. This study used the linked SEER‐Medicare database. The interpretation and reporting of these data are the sole responsibility of the authors. The authors acknowledge the efforts of the National Cancer Institute; the Office of Research, Development and Information, CMS; Information Management Services, Inc.; and the SEER Program tumor registries in the creation of the SEER‐Medicare database.
