# Supplementary material for: Survival in elderly patients with breast cancer with and without autoimmune disease
Source: Cancer Med. 2023 Apr 26;12(12):13086–99. doi: 10.1002/cam4.5989 (PMC10315719; doi:10.1002/cam4.5989)
Supplement: Supplementary file 1 — Data S1: [file CAM4-12-13086-s001.pdf]

Supplement:

Supplement 1: ICD-9 Diagnosis Codes for Identification of Autoimmune Disease

**Autoimmune Disease**	**ICD-9 Diagnosis Code**
Crohn's Disease	555, 555.1, 555.2, 555.3, 555.8, 555.9
Dermatomyositis	710.3
Polymyositis	710.4
Psoriasis	696, 696.1, 696.2
Rheumatoid Arthritis	714, 714.1, 714.2
Sarcoidosis	135
Systemic Scleroderma	710.1
Sjogren's Syndrome	710.2
Systemic Lupus Erythematosus	710
Ulcerative Colitis	556.5,556.6,556.8, 556.9

Supplement 2: ICD-10 Codes to Identify Patients who died of their Cancer

**ICD-10 Codes**
General Codes: C798, C80, C97, D489<br>Site Specific Codes: C32-C34, C39, C780, D022, D143, D144, D15, D38

Supplement 3: Commonly Used Chemotherapy Agents for the Treatment of Breast Cancer

**Agent**	**Healthcare Common Procedure Coding System Codes**
Docetaxel	J9171, J9170
Paclitaxel	C9127, C9431, J9264, J9265, J9267
Doxorubicin	J9000, C9415, J9002, Q2048, Q2049, Q2050, J9001
Gemcitabine	J9201

Supplement 4A: Univariate and Multivariable Cox Regression Analysis on OS (Stage I Patients)

	Univariate analysis		Multivariable analysis	
**Variables**	**HR (95% CI)**	**p-value**	**HR (95% CI)**	**p-value**
Autoimmune disease (No vs. Yes)	0.73 (0.7, 0.77)	< 0.0001	1.05 (1.01, 1.1)	0.025
Age (per year increase)	1.12 (1.11, 1.12)	< 0.0001	1.09 (1.09, 1.1)	< 0.0001
Race				
Black vs. White	1.16 (1.08, 1.25)	< 0.0001	1.33 (1.23, 1.43)	< 0.0001
Others vs. White	0.47 (0.41, 0.52)	< 0.0001	0.58 (0.51, 0.65)	< 0.0001
CKD (No vs. Yes)	0.17 (0.16, 0.18)	< 0.0001	0.35 (0.33, 0.36)	< 0.0001
Charlson comorbidity index (per index increase)	1.68 (1.67, 1.7)	< 0.0001		
NCI comorbidity index (per index increase)	1.61 (1.59, 1.62)	< 0.0001		
Chemotherapy (No vs. Yes)	0.52 (0.47, 0.56)	< 0.0001	0.49 (0.45, 0.55)	< 0.0001
Radiation (No vs. Yes)	2.26 (2.16, 2.36)	< 0.0001	1.66 (1.58, 1.73)	< 0.0001

CI, confidence interval; HR, hazard ratio; OS, overall survival.

Supplement 4B: Proportional Hazards Regression with Death as the Competing Risk on CSM (Stage I Patients)

	Univariate analysis		Multivariable analysis	
**Variables**	**HR (95% CI)**	**p-value**	**HR (95% CI)**	**p-value**
Autoimmune disease (No vs. Yes)	0.97 (0.86, 1.08)	0.541	1.35 (1.2, 1.51)	< 0.0001
Age (per year increase)	1.04 (1.04, 1.05)	< 0.0001	1.04 (1.03, 1.05)	< 0.0001
Race				
Black vs. White	1.78 (1.53, 2.06)	< 0.0001	1.73 (1.49, 2.01)	< 0.0001
Others vs. White	0.68 (0.53, 0.88)	0.003	0.79 (0.61, 1.01)	0.059
CKD (No vs. Yes)	0.39 (0.35, 0.44)	< 0.0001	0.58 (0.51, 0.66)	< 0.0001
Charlson comorbidity index (per index increase)	1.31 (1.26, 1.35)	< 0.0001		
NCI comorbidity index (per index increase)	1.26 (1.22, 1.3)	< 0.0001		
Chemotherapy (No vs. Yes)	0.11 (0.09, 0.12)	< 0.0001	0.11 (0.09, 0.12)	< 0.0001
Radiation (No vs. Yes)	1.74 (1.56, 1.93)	< 0.0001	1.53 (1.37, 1.71)	< 0.0001

CI, confidence interval; CSM, cancer specific mortality; HR, hazard ratio.

Supplement 5A: Univariate and Multivariable Cox Regression Analysis on OS (Stage II Patients)

	**Univariate analysis**		**Multivariable analysis**	
**Variables**	**HR (95% CI)**	**p-value**	**HR (95% CI)**	**p-value**
Autoimmune disease (No vs. Yes)	0.85 (0.81, 0.9)	< 0.0001	1.24 (1.18, 1.31)	< 0.0001
Age (per year increase)	1.08 (1.07, 1.08)	< 0.0001	1.06 (1.06, 1.06)	< 0.0001
Race				
Black vs. White	1.14 (1.1, 1.22)	0.0001	1.34 (1.25, 1.43)	< 0.0001
Others vs. White	0.62 (0.55, 0.69)	< 0.0001	0.73 (0.66, 0.82)	< 0.0001
CKD (No vs. Yes)	0.24 (0.23, 0.25)	< 0.0001	0.39 (0.38, 0.4)	< 0.0001
Charlson comorbidity index (per index increase)	1.57 (1.56, 1.59)	< 0.0001		
NCI comorbidity index (per index increase)	1.49 (1.48, 1.51)	< 0.0001		
Chemotherapy (No vs. Yes)	0.7 (0.66, 0.74)	< 0.0001	0.77 (0.73, 0.82)	< 0.0001
Radiation (No vs. Yes)	1.99 (1.9, 2.09)	< 0.0001	1.68 (1.6, 1.77)	< 0.0001

CI, confidence interval; HR, hazard ratio; OS, overall survival.

Supplement 5B: Proportional Hazards Regression with Death as the Competing Risk on CSM (Stage II Patients)

	**Univariate analysis**		**Multivariable analysis**	
**Variables**	**HR (95% CI)**	**p-value**	**HR (95% CI)**	**p-value**
Autoimmune disease (No vs. Yes)	1.01 (0.94, 1.09)	0.765	1.36 (1.26, 1.47)	< 0.0001
Age (per year increase)	1.02 (1.02, 1.02)	< 0.0001	1.01 (1.01, 1.02)	< 0.0001
Race				
Black vs. White	1.46 (1.33, 1.6)	< 0.0001	1.43 (1.3, 1.58)	< 0.0001
Others vs. White	0.71 (0.6, 0.83)	< 0.0001	0.75 (0.64, 0.88)	0.0003
CKD (No vs. Yes)	0.51 (0.48, 0.55)	< 0.0001	0.64 (0.59, 0.7)	< 0.0001
Charlson comorbidity index (per index increase)	1.21 (1.18, 1.24)	< 0.0001		
NCI comorbidity index (per index increase)	1.18 (1.16, 1.2)	< 0.0001		
Chemotherapy (No vs. Yes)	0.39 (0.36, 0.41)	< 0.0001	0.39 (0.36, 0.42)	< 0.0001
Radiation (No vs. Yes)	1.52 (1.41, 1.63)	< 0.0001	1,45 (1.35, 1.55)	< 0.0001

CI, confidence interval; CSM, cancer specific mortality; HR, hazard ratio.

Supplement 6A: Univariate and Multivariable Cox Regression Analysis on OS (Stage III Patients)

	**Univariate analysis**		**Multivariable analysis**	
**Variables**	**HR (95% CI)**	**p-value**	**HR (95% CI)**	**p-value**
Autoimmune disease (No vs. Yes)	0.93 (0.87, 0.99)	0.027	1.34 (1.25, 1.43)	< 0.0001
Age (per year increase)	1.05 (1.04, 1.05)	< 0.0001	1.03 (1.03, 1.04)	< 0.0001
Race				
Black vs. White	1.28 (1.18, 1.37)	< 0.0001	1.42 (1.32, 1.54)	< 0.0001
Others vs. White	0.78 (0.68, 0.88)	0.0001	0.86 (0.75, 0.97)	0.018
CKD (No vs. Yes)	0.33 (0.31, 0.35)	< 0.0001	0.47 (0.44, 0.5)	< 0.0001
Charlson comorbidity index (per index increase)	1.44 (1.42, 1.47)	< 0.0001		
NCI comorbidity index (per index increase)	1.4 (1.38, 1.42)	< 0.0001		
Chemotherapy (No vs. Yes)	0.61 (0.58, 0.65)	< 0.0001	0.79 (0.74, 0.84)	< 0.0001
Radiation (No vs. Yes)	1.86 (1.76, 1.97)	< 0.0001	1.6 (1.51, 1.69)	< 0.0001

CI, confidence interval; HR, hazard ratio; OS, overall survival.

Supplement 6B: Proportional Hazards Regression with Death as the Competing Risk on CSM (Stage III Patients)

	**Univariate analysis**		**Multivariable analysis**	
**Variables**	**HR (95% CI)**	**p-value**	**HR (95% CI)**	**p-value**
Autoimmune disease (No vs. Yes)	1.07 (0.99, 1.15)	0.107	1.41 (1.29, 1.53)	< 0.0001
Age (per year increase)	1.02 (1.02, 1.02)	< 0.0001	1.01 (1.01, 1.01)	< 0.0001
Race				
Black vs. White	1.34 (1.23, 1.46)	< 0.0001	1.37 (1.25, 1.49)	< 0.0001
Others vs. White	0.87 (0.75, 1.01)	0.058	0.94 (0.81, 1.09)	0.423
CKD (No vs. Yes)	0.55 (0.51, 0.59)	< 0.0001	0.69 (0.63, 0.75)	< 0.0001
Charlson comorbidity index (per index increase)	1.18 (1.15, 1.21)	< 0.0001		
NCI comorbidity index (per index increase)	1.16 (1.13, 1.19)	< 0.0001		
Chemotherapy (No vs. Yes)	0.53 (0.49, 0.56)	< 0.0001	0.55 (0.51, 0.59)	< 0.0001
Radiation (No vs. Yes)	1.53 (1.43, 1.64)	< 0.0001	1.44 (1.35, 1.54)	< 0.0001

CI, confidence interval; CSM, cancer specific mortality; HR, hazard ratio.

Supplement 7A: Univariate and Multivariable Cox Regression Analysis on OS (Stage IV Patients)

	**Univariate analysis**		**Multivariable analysis**	
**Variables**	**HR (95% CI)**	**p-value**	**HR (95% CI)**	**p-value**
Autoimmune disease (No vs. Yes)	1.14 (1.07, 1.22)	0.0001	1.45 (1.35, 1.55)	< 0.0001
Age (per year increase)	1.04 (1.035, 1.04)	< 0.0001	1.036 (1.03, 1.04)	< 0.0001
Race				
Black vs. White	1.13 (1.05, 1.22)	0.001	1.32 (1.23, 1.43)	< 0.0001
Others vs. White	0.64 (0.56, 0.73)	< 0.0001	0.72 (0.63, 0.82)	< 0.0001
CKD (No vs. Yes)	0.6 (0.57, 0.64)	< 0.0001	0.76 (0.71, 0.8)	< 0.0001
Charlson comorbidity index (per index increase)	1.31 (1.28, 1.33)	< 0.0001		
NCI comorbidity index (per index increase)	1.25 (1.23, 1.27)	< 0.0001		
Chemotherapy (No vs. Yes)	1.01 (0.95, 1.07)	0.723	1.06 (1, 1.12)	0.063
Radiation (No vs. Yes)	1.43 (1.35, 1.52)	< 0.0001	1.26 (1.19, 1.34)	< 0.0001

CI, confidence interval; HR, hazard ratio; OS, overall survival.

Supplement 7B: Proportional Hazards Regression with Death as the Competing Risk on CSM (Stage IV Patients)

	**Univariate analysis**		**Multivariable analysis**	
**Variables**	**HR (95% CI)**	**p-value**	**HR (95% CI)**	**p-value**
Autoimmune disease (No vs. Yes)	1.2 (1.11, 1.28)	< 0.0001	1.4 (1.29, 1.5)	< 0.0001
Age (per year increase)	1.02 (1.022, 1.03)	< 0.0001	1.02 (1, 1.03)	< 0.0001
Race				
Black vs. White	1.09 (1.01, 1.18)	0.031	1.2 (1.11, 1.31)	< 0.0001
Others vs. White	0.65 (0.56, 0.74)	< 0.0001	0.91 (0.62, 0.81)	< 0.0001
CKD (No vs. Yes)	0.74 (0.69, 0.79)	< 0.0001	0.88 (0.82, 0.94)	0.0002
Charlson comorbidity index (per index increase)	1.11 (1.07, 1.14)	< 0.0001		
NCI comorbidity index (per index increase)	1.09 (1.06, 1.12)	< 0.0001		
Chemotherapy (No vs. Yes)	0.95 (0.9, 1)	0.058	0.94 (0.89, 1)	0.048
Radiation (No vs. Yes)	1.26 (1.19, 1.34)	< 0.0001	1.13 (1.1, 1.21)	< 0.0001

CI, confidence interval; CSM, cancer specific mortality; HR, hazard ratio.
